# Growth velocity in children with Environmental Enteric Dysfunction is associated with specific bacterial and viral taxa of the gastrointestinal tract in Malawian children

**DOI:** 10.1371/journal.pntd.0008387

**Published:** 2020-06-23

**Authors:** Chandni Desai, Scott A. Handley, Rachel Rodgers, Cynthia Rodriguez, Maria I. Ordiz, Mark J. Manary, Lori R. Holtz

**Affiliations:** 1 Department of Pathology and Immunology, Washington University School of Medicine, St. Louis, Missouri, United States of America; 2 Edison Family Center for Genome Sciences & Systems Biology, Washington University School of Medicine, St. Louis, Missouri, United States of America; 3 Department of Pediatrics, Washington University School of Medicine, St. Louis, Missouri, United States of America; 4 Department of Pediatrics, College of Medicine, University of Malawi, Blantyre, Malawi; University of Texas Medical Branch, UNITED STATES

## Abstract

Environmental enteric dysfunction (EED) is characterized by diffuse villous atrophy of the small bowel. EED is strongly associated with stunting, a major public health problem linked to increased childhood morbidity and mortality. EED and subsequent stunting of linear growth are surmised to have microbial origins. To interrogate this relationship, we defined the comprehensive virome (eukaryotic virus and bacteriophage) and bacterial microbiome of a longitudinal cohort of rural Malawian children with extensive metadata and intestinal permeability testing at each time point. We found thirty bacterial taxa differentially associated with linear growth. We detected many eukaryotic viruses. Neither the total number of eukaryotic families nor a specific viral family was statistically associated with improved linear growth. We identified 3 differentially abundant bacteriophage among growth velocities. Interestingly, there was a positive correlation between bacteria and bacteriophage richness in children with subsequent adequate/moderate growth which children with subsequent poor growth lacked. This suggests that a disruption in the equilibrium between bacteria and bacteriophage communities might be associated with subsequent poor growth. Future studies of EED and stunting should include the evaluation of viral communities in addition to bacterial microbiota to understand the complete microbial ecology of these poorly understood entities.

## Author summary

Stunting (poor linear growth) is a major global problem. Stunting affects one-third of the half-billion preschool children in low and middle-income countries and is associated with ~20% of all-cause deaths before age five. Stunting is believed to be a consequence of environmental enteric dysfunction (EED). EED is a gut inflammatory process that is endemic in children living in low and middle-income countries. EED and, by extension, stunting are thought to have microbial origins. However, attempts to find specific pathogens that drive (or protect from) EED have not been fruitful. Here, we define the comprehensive gut virome, which includes viruses that infect humans and animals and viruses that infect bacteria (bacteriophage) and the bacterial gut microbiome in rural Malawian children. These participants are very well characterized (with careful growth measurements and intestinal permeability testing), enabling us to more precisely correlate the populations of viruses and bacteria in their stool with how well, or how poorly, the children are growing. We found thirty bacterial species and three bacteriophage that were differentially associated with linear growth in the three months after sampling. We found a positive correlation between bacteria and bacteriophage richness in children with subsequent adequate and moderate linear growth which children with subsequent poor growth lacked. Our data suggest that disrupting the bacteria-bacteriophage equilibrium between bacteria and bacteriophage communities might impair childhood linear growth.

## Introduction

Stunting, defined as height for age greater than two standard deviations below the median, is a neglected tropical disease. One-third of the half-billion preschool children in low and middle-income countries are stunted [1], an anthropometric status that is strongly associated with ~20% of all-cause mortality before age five. Stunted individuals who survive early childhood are predisposed to long-term cognitive deficits, poor school performance, lower economic productivity [1], and metabolic syndrome [2]. Stunting has intergenerational consequences, since maternal short stature, presumably a legacy of childhood stunting, is a risk for intra-uterine growth retardation [3] and perinatal mortality [4].

Environmental enteric dysfunction (EED) was first described in healthy adults in resource poor settings [5], then in Peace Corps volunteers in South Asia [6], and later in children in regions of the world with inadequate dietary intake, sanitation and hygiene [7]. EED is characterized by diffuse villous atrophy of the small bowel. EED has been defined through biopsies: decreased villus height, lymphocytic infiltration of the lamina propria, and increased density of intraepithelial lymphocytes. EED is also associated with increased small bowel permeability measured using sugar testing (i.e. lactulose excretion), and is typically silent. EED is strongly associated with stunting, a major public health problem linked to increased morbidity and childhood mortality [8, 9].

It is surmised that EED is related to the environment because of its geographic distribution. Furthermore, when expatriates develop EED in endemic areas, small bowel lesions resolve on repatriation to North America and Europe [10, 11]. It is plausible that EED and, by extension stunting, have microbial origins, especially as nutritional interventions fail to correct most of the defect in stature in stunted children [12].

Recent studies have suggested that intestinal bacterial communities can affect childhood linear growth [13, 14]. However, we know little about the impact of viral communities (eukaryotic or phage) or of specific viruses on linear growth. Such an association would be plausible: viruses can compromise intestinal mucosal integrity, [15] and enteric viruses (rotavirus, astrovirus, norovirus, and enteroviruses) replicate in the human small bowel [16–18]. Additionally, children from resource-poor regions have much more diverse intestinal eukaryotic viromes than those from high-income settings [19]. In addition to these geographic differences, a study of fecal transcripts from children with EED showed an increased expression of messages reflecting host response to viral infection [20]. These data support the concept that viruses drive the development or the perpetuation of EED and, by extension, stunting.

Even less is known about the role of bacteriophages in disease. Decreased bacterial diversity has been associated with disorders such as inflammatory bowel diseases, and it is postulated that bacteriophage decrease bacterial diversity [21, 22].

Here, we define the comprehensive virome (eukaryotic virus and bacteriophage) and bacterial microbiome of a longitudinal cohort of rural Malawian children with extensive metadata and intestinal permeability testing at each time point, to examine the inter-relationships between these categories of data. Specifically, we seek a viral and/or bacterial signature of poor growth velocity in Malawian children with EED. To do so, we examined if viral or bacterial communities or individual taxa differ in children with EED across growth velocity categories.

## Methods

### Ethics statement

Verbal and written consent was obtained from the primary caregiver of each child to participate in the parent study [23]. This study was approved by the Human Research Protection Office of Washington University School of Medicine in St. Louis (approval 201706145).

### Subjects

Forty-nine children without diarrhea or HIV were randomly selected from the parent study, [23] which enrolled all eligible children aged 12 to 35 months old in a 9-village cluster of Nthenda in the Chikwawa district of southern Malawi. Children in this area live in mud huts and the maize-based diet is largely uniform throughout the community. Therefore, this subset likely represents environmental conditions typical of rural southern African children.

**Fig 1:**
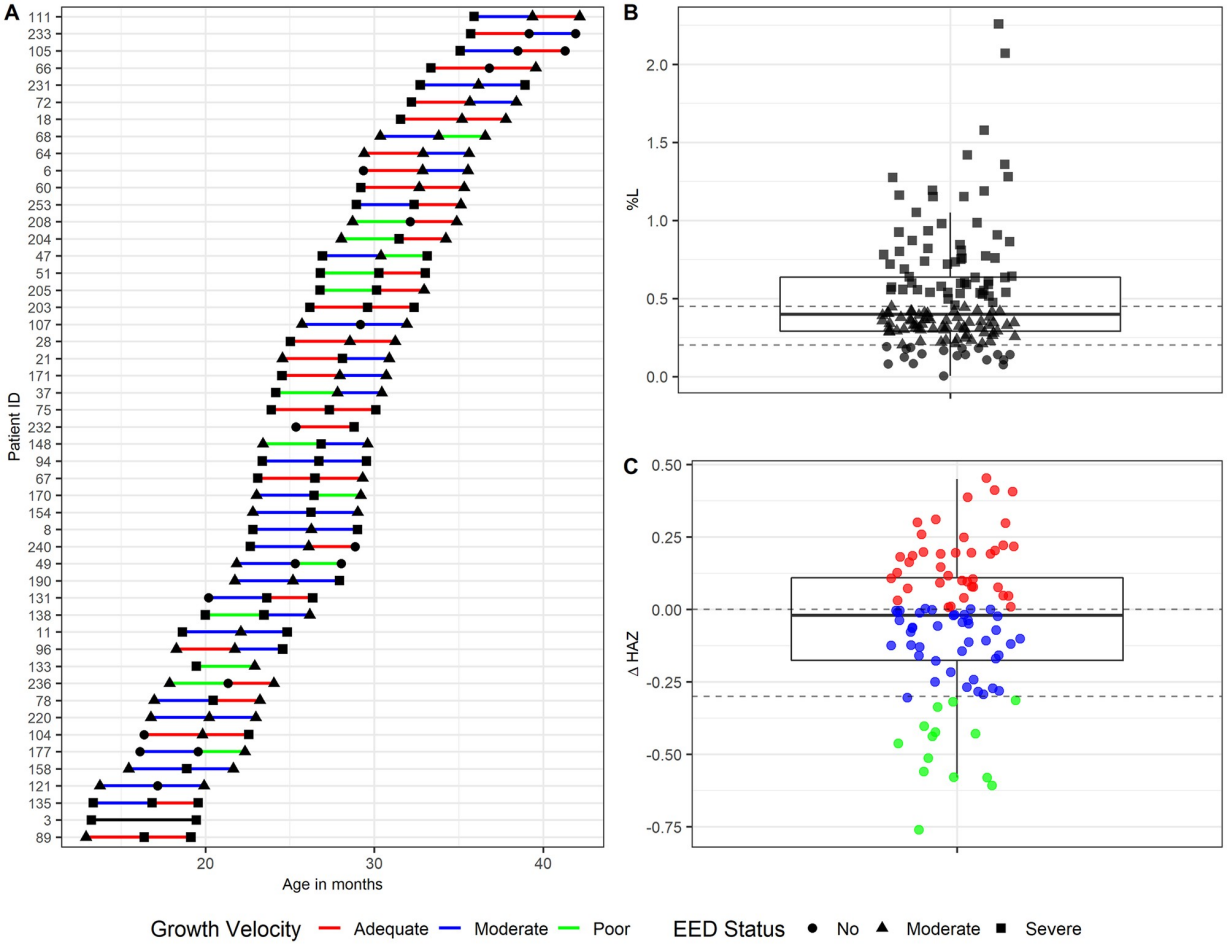
Overall cohort description. A) Age of sample collection for each participant during the course of the study. The colored lines denote growth velocity in each time period. The shape of the symbol represents the participant’s EED status based on %L. B) Distribution of %L measurements (three time points for all subjects (n = 49). The two horizontal lines denote enteropathy status (%L<0.2: no disease; %L>0.45: severe disease). C) Distribution of the difference of height for age Z score (HAZ) between time points from the same child. The two horizontal lines denote growth velocity over a 3-month period (delta HAZ > 0: Adequate growth, delta HAZ< -0.3: Poor growth).

Stools were collected at enrollment, and 3 months and 6 months later (Fig 1A), snap frozen in polypropylene vials in liquid nitrogen within minutes of collection, and stored at -80°C until processing. At each time point, dual sugar absorption testing with lactulose and mannitol was performed as previously described [23]. Metadata collected included anthropometric measurements (weight, length, and midupper arm circumference), food insecurity questionnaires, diet history, and symptom surveys.

To define children with EED, we used % lactulose (%L) cut points of <0.2%, ≥0.2% and ≤0.45%, and >0.45% L to signify children with no, moderate, and severe EED as we have previously reported [24]. Growth velocity was defined as the difference between height for age Z score (HAZ) between time points. We defined growth velocities as adequate, moderate, and poor if the ΔHAZ over an interval was >0, ≤0 and ≥ -0.3, and <-0.3, respectively [25]. Stool analysis was limited to samples from children displaying evidence of moderate or severe EED at sampling time. For each three-month interval of analysis, we used the stool samples obtained at the beginning of the observation period to define the microbial community for that interval.

### Bacterial 16S rRNA gene amplicon sequencing

100 mg of stool was disrupted by bead beating and DNA was extracted using QIAamp DNA Stool Mini Kit on a QIAcube automated DNA extraction unit. PCR was performed using Golay-barcoded primers specific for the V4 region (F515/R806). Reactions were held at 94°C for 2 min to denature DNA, with amplification proceeding for 40 cycles at 94 °C for 15 s, 50 °C for 30 s, and 68 °C for 30 s; a final extension of 2 min at 68 °C. Samples were amplified in triplicate, combined, and cleaned using Agencourt Ampure XP (Beckman-Coulter) bead clean up kit. Equimolar libraries were pooled and sequenced using an Illumina MiSeq sequencer (2x250 v2 kit) at the Center for Genome Sciences & Systems Biology at Washington University.

### Bacterial 16S rRNA gene amplicon analysis

Read quality control and the resolution of amplicon sequence variants (ASVs) were performed with the dada2 R package [26]. ASVs that were not assigned to the kingdom Bacteria, or were assigned to the Family Mitochondria, the Class of Chloroplast or the Phylum of Cyanobacteria/Chloroplast, were filtered out. The remaining reads were assigned taxonomy using the Ribosomal Database Project (RDP trainset 16/release 11.5) 16S rRNA gene sequence database [27]. Analysis was performed on unrarefied data. All subsequent ecological analyses were performed using PhyloSeq and other R packages [28]. A fully reproducible workflow of the analysis presented in this manuscript can be found at (https://github.com/chandni177/BacterialViralMicrobiome_of_GrowthVelocity_in_EED) along with the required data files.

ASVs differentially abundant between growth velocity groups in children with moderate and severe EED were identified by performing three pairwise comparisons using DESeq2 [29]. For this analysis each ASV was treated as a unique entity. Differential abundance was determined using the Wald test with significance at p-adjusted value < 0.05. Taxa with a mean abundance of at least 10 across samples were selected for subsequent analysis. As part of the DESeq2 analysis, we accounted for repeated sampling and potential confounding of bacterial community composition by breastfeeding status by including these variables as part of the model.

### Virome sequencing

Chipped fecal specimens (approximately 200 mg) were diluted in phosphate-buffered saline (PBS) in a 1:6 ratio and filtered through a 0.45-μm-pore-size membrane. Total nucleic acid was extracted from the filtrate using COBAS Ampliprep (Roche). Sequence-independent DNA and RNA amplification (SIA) was performed on the total nucleic acid as previously described [30] and used to construct a NEBNext library (Illumina). Libraries were purified and size-selected using Agencourt Ampure XP beads (Beckman-Coulter), followed by quantification using a 2100 Bioanalyzer (Agilent Technologies). Equimolar libraries were pooled and sequenced using an Illumina MiSeq sequencer (2x250 v2 kit) at the Center for Genome Sciences & Systems Biology at Washington University.

### Virome analysis

Illumina MiSeq sequencing reads (2x250bp) were demultiplexed and adapter sequences were trimmed. Reads were analyzed using VirusSeeker [31], a BLAST-based computational pipeline to identify viral sequences. Bacteriophage taxonomies were assigned using the lowest-common ancestor algorithm implemented in MEGAN Community Edition (v6) [32] with the following parameters: Min Support: 1, Min Score: 40.0, Max Expected: 0.001, Top Percent: 10.0, Min-Complexity filter: 0.3, useMinimalCoverageHeuristic = true. Because of the presence of low complexity/repetitive regions in the reads, the following false-positive virus family taxonomic assignments were omitted from the analysis: *Herpesviridae*, *Maresilleviridae*, *Mimiviridae*, *Phycodnaviridae*, and *Poxviridae*. Ecological analyses were performed using PhyloSeq and additional R packages [28]. Viral taxa with fewer than three total reads were masked.

A presence-absence heatmap of eukaryotic viral families was generated for all growth velocity groups. We compared the average normalized abundance of the *Adenoviridae*, *Anelloviridae*, and the *Picornaviridae* genus of enterovirus across growth velocity groups.

Differentially abundant phage taxa between growth velocity among children with moderate and severe EED were identified by performing three pairwise comparisons using DESeq2. As part of the DESeq2 analysis, we also accounted for repeated sampling and potential confounding of viral community composition by breastfeeding status by including these variables as part of the model. Taxa present in fewer than two samples were excluded from the analysis. Significantly differentially abundant taxa were identified as those having a p-adjusted value < 0.1.

### Bacteriophage-bacteria interaction analysis

Spearman correlation coefficients were calculated between bacterial and bacteriophage richness and Shannon diversity for each growth velocity group. Using multiple linear regression with growth velocity as a covariate and accounting for repeated sampling, we tested the relationship between bacterial and bacteriophage richness and Shannon diversity. ANCOVA was used to determine if differences in the relationship between bacterial and bacteriophage richness and diversity can be explained by growth velocity group while accounting for repeated sampling. Spearman correlation was performed between the normalized abundances of bacterial ASVs and bacteriophage which were significantly associated with growth velocity as identified by DESeq2. These correlations were visualized in heatmaps.

### Statistics

Differences in alpha diversity (richness and Shannon diversity) and normalized abundance of specific taxa were evaluated between groups using Kruskal-Wallis and Dunn’s multiple comparisons. Using multiple linear regression we examined the relationship between alpha diversity and growth velocity as a continuous variable while accounting for repeated sampling and controlling for age, baseline HAZ, gender, and breastfeeding status. Differences in beta-diversity were determined using Permutational Multivariate Analysis of Variance (ADONIS). The relationship between viral and bacterial richness and age was assessed with linear regressions. The bacteria-bacteriophage richness and diversity analysis multiple linear regression analysis was performed using the lm() function in R. All p-values are two-tailed.

## Results

### Description of the cohort

The age of the children at the time of sampling ranged from 12–43 months (Fig 1A and Table 1). The majority of samples were from children with either moderate or severe EED at the time of sampling (Fig 1B). The distribution of ΔHAZ of the cohort is shown in Fig 1C. %L did not predict subsequent ΔHAZ by linear regression (p = 0.81). Other characteristics of the cohort are summarized in Table 1. Because diet is also known to determine bacterial community content in the gut [33, 34] and possibly also viral [35, 36] community composition, we examined at what age children stopped breastfeeding because the main diet in this population otherwise has little variation. All children breastfed initially, and the median age of breastfeeding cessation did not significantly vary according to growth status (Table 1).

**Table 1:** Demographics of cohort.

	Poor growth velocity and EED (n = 13)	Moderate growth velocity and EED (n = 37)	Adequate growth velocity and EED (n = 34)
Age (median, Q1, Q3) (mos.)	26.4 (20.0, 28.0)	25.2 (21.7, 28.1)	26.9 (23.2, 31.1)
Male (%)	69.0%	59.5%	56%
Weight for height Z score (median, Q1, Q3)	-0.23 (-1.0, 0.5)	0.02 (-0.8, 0.9)	-0.17 (-0.6, 0.2)
Height for age Z score (median, Q1, Q3)	-1.7 (-3.1, -0.8)	-1.4 (-2.0, -0.8)	-2.2 (-3.0, -1.7)
Age of breastfeeding cessation (median, Q1, Q3)	21.5 (20.0, 23.0)	24.0 (23.0, 26.0)	23.0 (20.0, 25.0)
ΔHAZ (median, Q1, Q3)	-0.44 (-0.58,-0.42)	-0.11(-0.17,-0.02)	0.14 (0.08, 0.22)
Parent study intervention			
Micronutrient & Fish oil (%)	31%	30%	32%
Micronutrient (%)	46%	43%	47%
Placebo (%)	23%	27%	21%

### Bacterial community structure does not differ by growth velocity in children with EED

**Fig 2:**
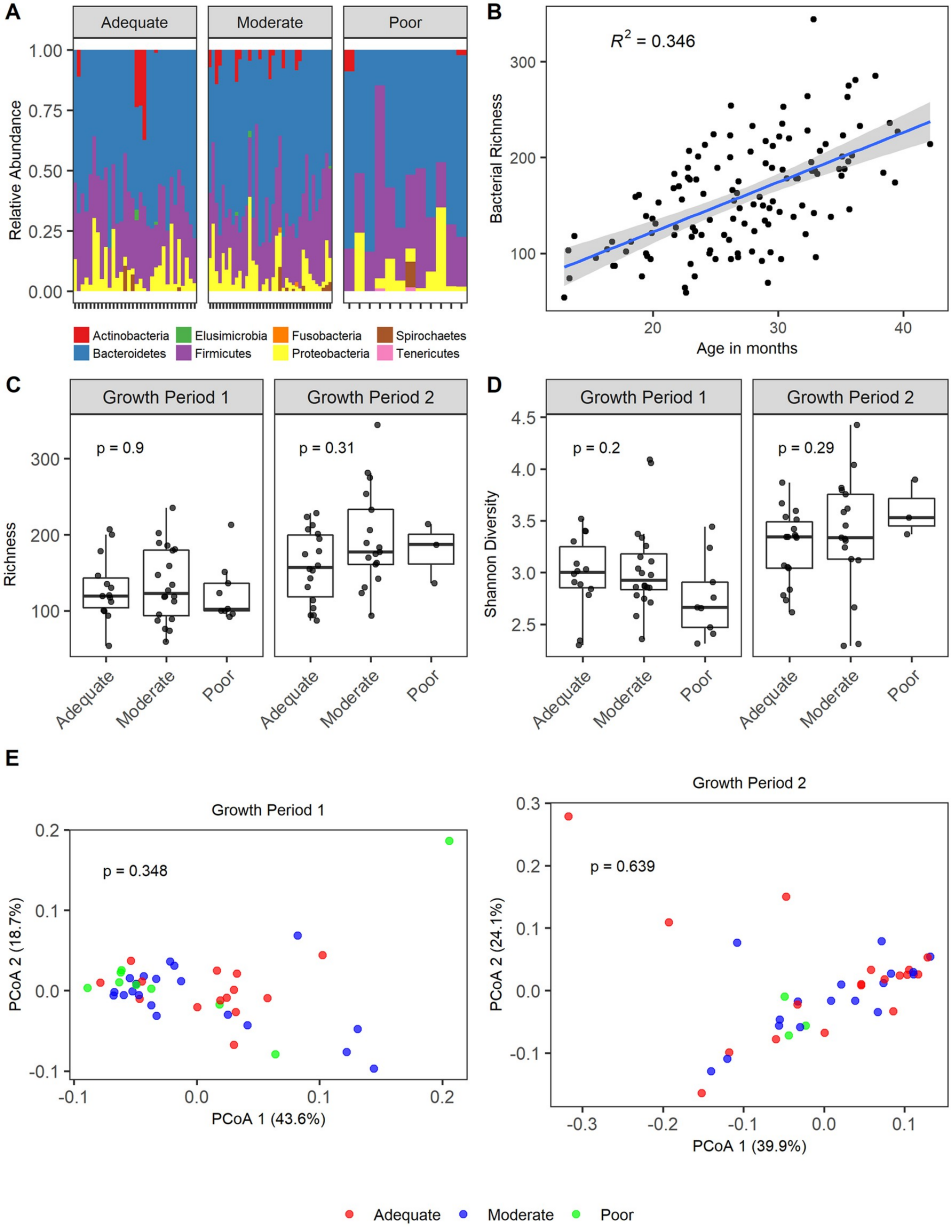
Composition of the bacterial microbiome of growth faltering in Environmental Enteric Dysfunction. A) Relative abundance of bacterial phyla in stools of children with moderate and severe EED within each growth velocity group (adequate growth velocity: ΔHAZ >0, moderate growth velocity: ΔHAZ ≤0 and ≥ -0.3, poor growth velocity ΔHAZ < -0.3) prior to the growth period. B) Bacterial richness over age. Linear regression, R^2^ value and 95% confidence intervals are shown. C) Bacterial richness of stool samples obtained at the beginning of a growth interval. Statistical significance assessed by Kruskal-Wallis. D) Shannon diversity of stool samples obtained at the beginning of a growth interval. Statistical significance assessed by Kruskal-Wallis. E) PCoA plot of weighted UniFrac distances. Statistical significance assessed by ADONIS.

16S rRNA gene sequencing generated an average of 53078 reads per sample (s.d 14286). Overall there were similar community patterns among the children with EED across the three growth velocity groups. The predominant phyla were Bacteroidetes, Firmicutes, and Proteobacteria (Fig 2A). Because of the reported relationship between the bacterial microbiome [37] we examined bacterial richness (Fig 2B) across age in all samples. As expected [37], we found that bacterial richness did increase with age, but the rate of increase did not differ by growth status. Potential confounding by age is lessened in this analysis because the median age of children did not vary with growth status (Table 1). We found no significant difference between the bacterial richness and Shannon diversity of samples from children with EED and across growth velocity groups (Fig 2C and 2D). Data from each growth period was analyzed independently to avoid repeated sampling. To examine the relationship between alpha diversity (richness and Shannon diversity) and growth velocity as a continuous variable (instead of categorical) while accounting for repeated measures and covariates (age, breastfeeding status, baseline HAZ, and gender), we performed multiple linear regression, and found no significance (richness p = 0.30; Shannon diversity p = 0.18). Furthermore, principal coordinate analysis (PCoA) of weighted UniFrac distances showed no significant differences in the beta-diversity of samples from children across growth velocities (Fig 2E).

### Bacterial taxa differentiate growth velocity status in children with moderate-severe EED

**Fig 3:**
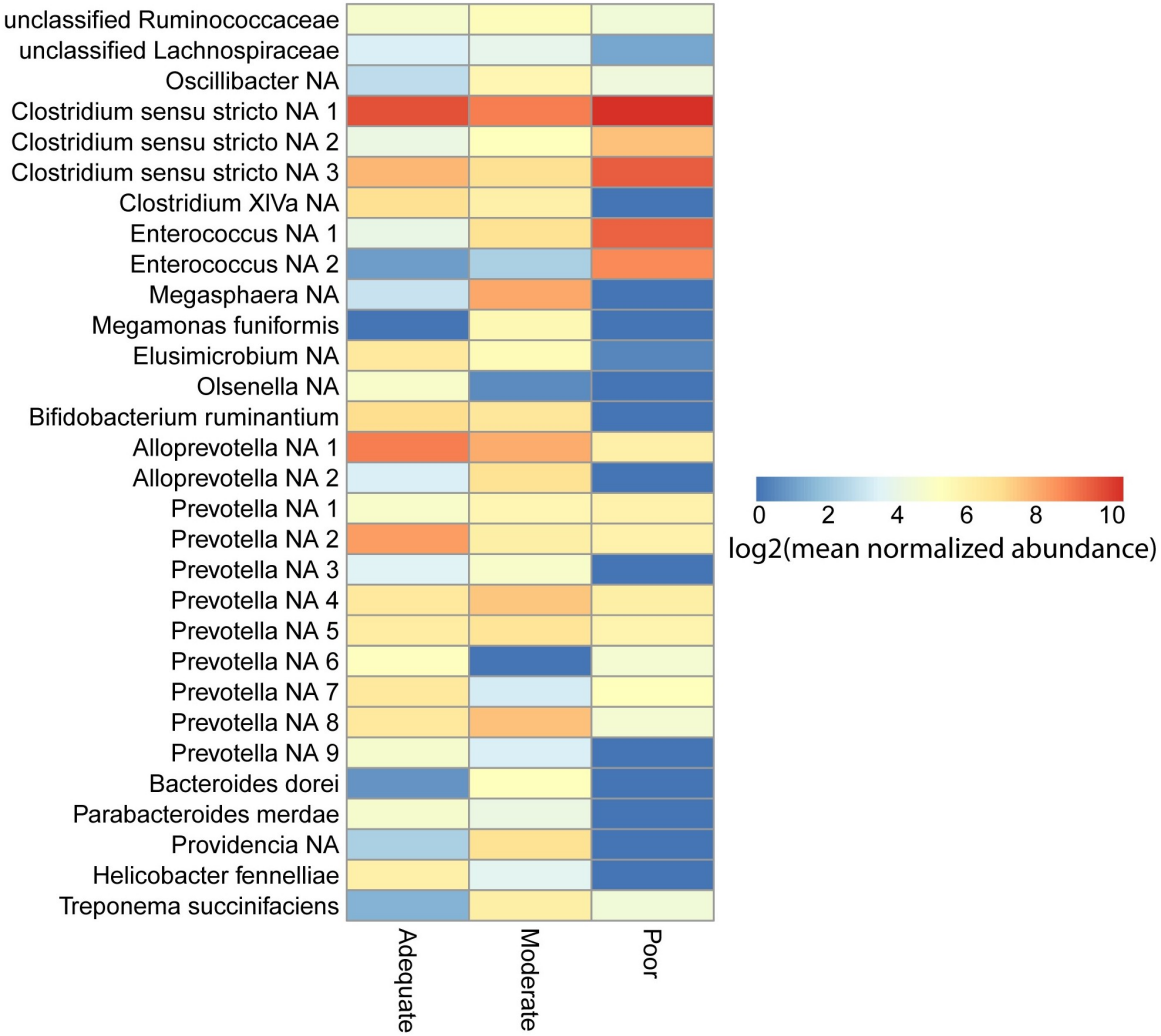
Bacterial taxa differentially abundant by growth velocity among children with EED. Bacterial taxa significantly differentially abundant across growth velocity groups colored by log2 mean normalized abundance.

We next considered the possibility that although the bacterial community structure was similar among children with different growth velocities, specific bacterial taxa might distinguish growth velocity in the subsequent three months. Thirty bacterial ASVs were significantly differentially abundant (S1 Table). We next examined the normalized abundances of these differentially abundant ASVs (Fig 3). DESeq2 selects taxa based on either a difference in mean abundance, the presence or absence of a taxa, or a combination of these factors. Notably, 10 taxa (*Megasphera* NA, *Helicobacter fennelliae*, *Clostridium* XIVa, *Olsenella*, *Prevotella* NA (two), *Bacteroides dorei*, *Parabacteroides merdae*, *Providencia* NA, and *Bifidobacterium ruminantium)* were present only in samples from children with subsequent adequate and moderate growth velocity and are completely absent in samples from children with poor growth.

### Eukaryotic virome of children does not differ by growth velocity in children with EED

**Fig 4:**
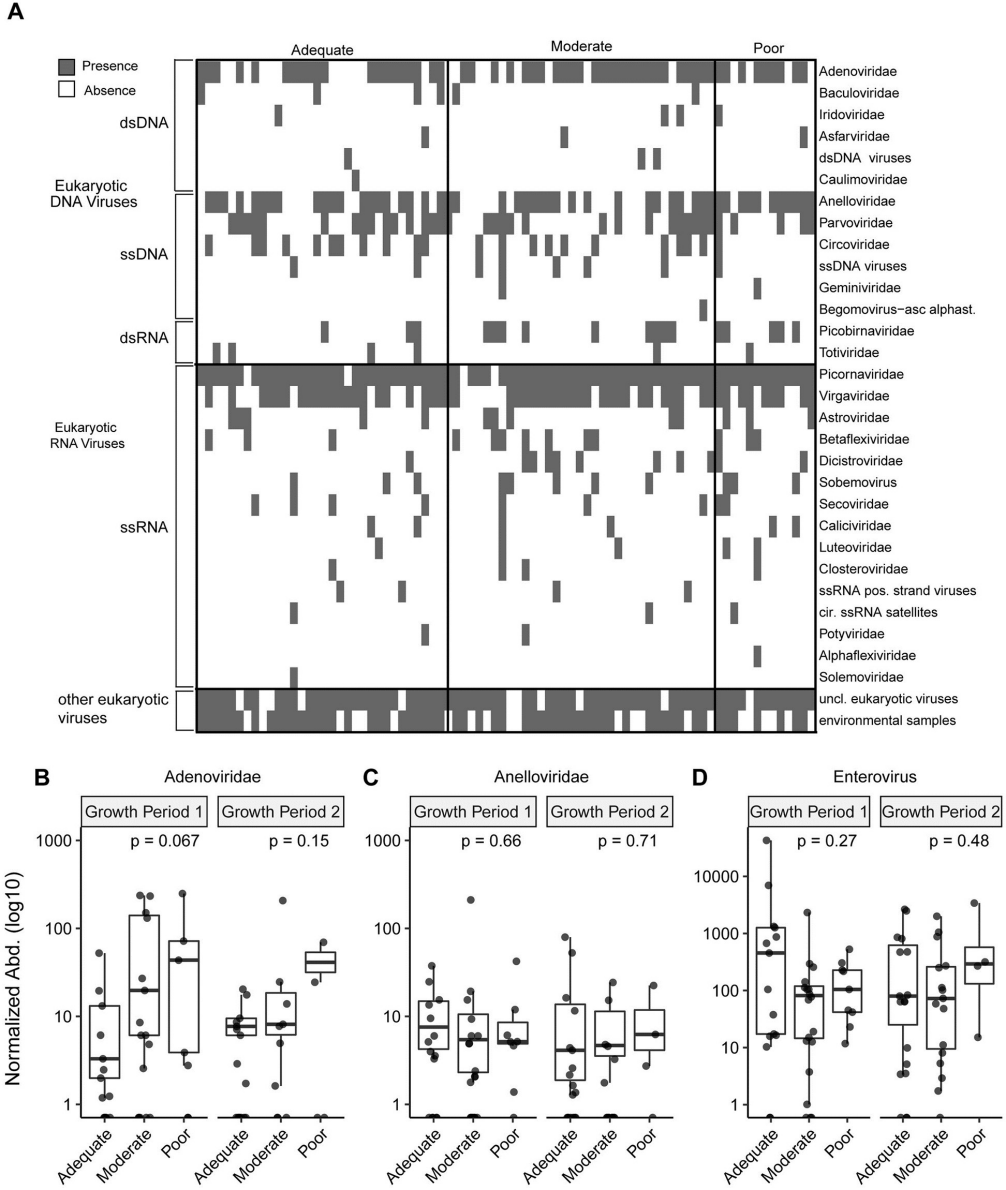
Composition of eukaryotic virome of growth faltering in Evironmental Enteric Dysfunction. A) Presence and absence heatmap of eukaryotic viral families in children with moderate-severe EED grouped by growth velocity status. Normalized abundance of frequently detected eukaryotic viruses in stool samples obtained at the beginning of a growth interval: B) *Adenoviridae*, C) *Anelloviridae*, and D) Enterovirus. Statistical significance assessed by Kruskal-Wallis.

We obtained an average of 1,328,312 (s.d. 741,157) reads per sample library. The read depth was not statistically different between growth velocity groups. We identified multiple eukaryotic DNA and RNA viruses in children with moderate and severe EED (Fig 4A). The most frequently detected DNA and RNA viral families were *Adenoviridae* and *Anelloviridae*, *and Picornaviridae* (genus Enterovirus) and *Virgaviridae*, respectively. The number of viral families detected did not statistically differ by growth velocity group. The average normalized abundance of *Adenoviridae*, *Anelloviridae*, and enteroviruses did not differ significantly across growth categories (Fig 4B, 4C and 4D). Additionally, the presence or absence of these viral taxa was not associated with subsequent poor or adequate growth.

### More diverse phageome in children EED with adequate growth

**Fig 5:**
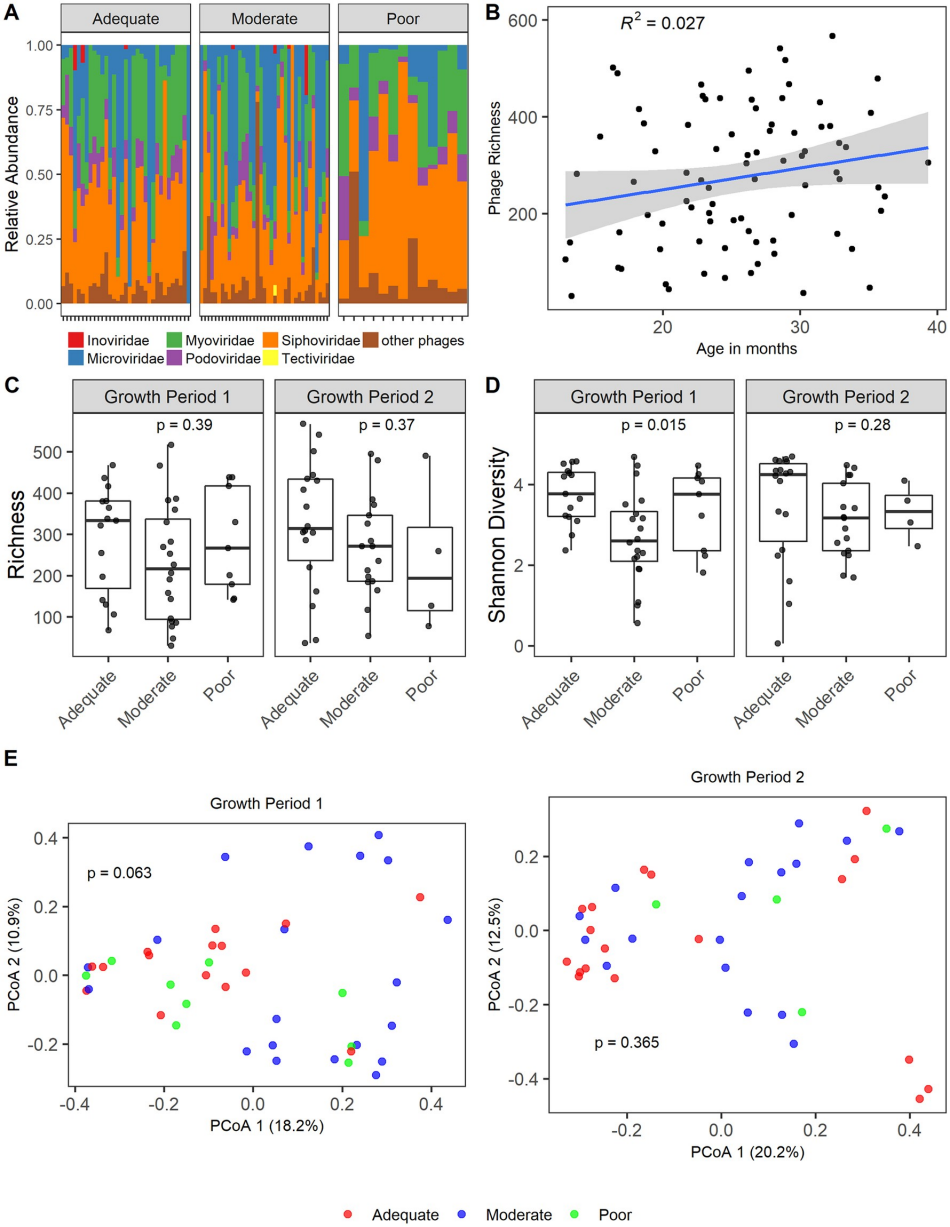
Composition of phageome of growth faltering in Environmental Enteric Dysfunction. A) Relative abundance of bacteriophage in stools of children with moderate and severe EED within each growth velocity group (adequate growth velocity: ΔHAZ >0, moderate growth velocity: ΔHAZ ≤0 and ≥ -0.3, poor growth velocity ΔHAZ <-0.3) prior to the growth period. B) Viral richness with age. R^2^ value and 95% confidence intervals are shown. C) Phage richness of stool samples obtained at the beginning of a growth interval. Statistical significance assessed by Kruskal-Wallis. D) Shannon diversity of stool samples obtained at the beginning of a growth interval. Statistical significance assessed by Kruskal-Wallis. E) PCoA plot of Bray Curtis distances. Statistical significance assessed by ADONIS.

We identified overall similar phage community patterns among children with EED across the different growth velocity groups (Fig 5A). The most abundant bacteriophages were from the *Caudovirales* order (*Siphoviridae*, *Inoviridae*, *Myoviridae* and *Podoviridae* families) and the *Microviridae* family. Because of the reported relationship between the virome with age [30], we examined viral richness (Fig 5B) across age. In comparison to the bacterial microbiome, viral richness was more stable across age. This is in contrast to what has previously been described in a North American birth cohort followed to age 2 years [30]. Phage richness did not significantly differ in children with EED across growth velocity groups (Fig 5C). In stools obtained prior to the first growth interval phage diversity (Fig 5D) was greater in those children with subsequent adequate growth than children with moderate growth (Kruskal-Wallis p = 0.015, Dunn’s Correction p = 0.012). However, the lack of significant differences in phage diversity in stools obtained before the second growth interval and the lack of a gradation with disease severity makes the biological relevance of this finding less clear. To test the relationship between alpha diversity and growth velocity (continuous variable) while accounting for repeated measures, age, breastfeeding status, baseline HAZ, and gender we performed multiple linear regression, and found no significance (richness p = 0.27; Shannon diversity p = 0.25). Principal coordinate analysis (PCoA) of Bray-Curtis distances demonstrated no significant differences in the beta-diversity of phage in samples from children across growth velocities (Fig 5E).

### Bacteriophage differentiate growth status in EED

**Fig 6:**
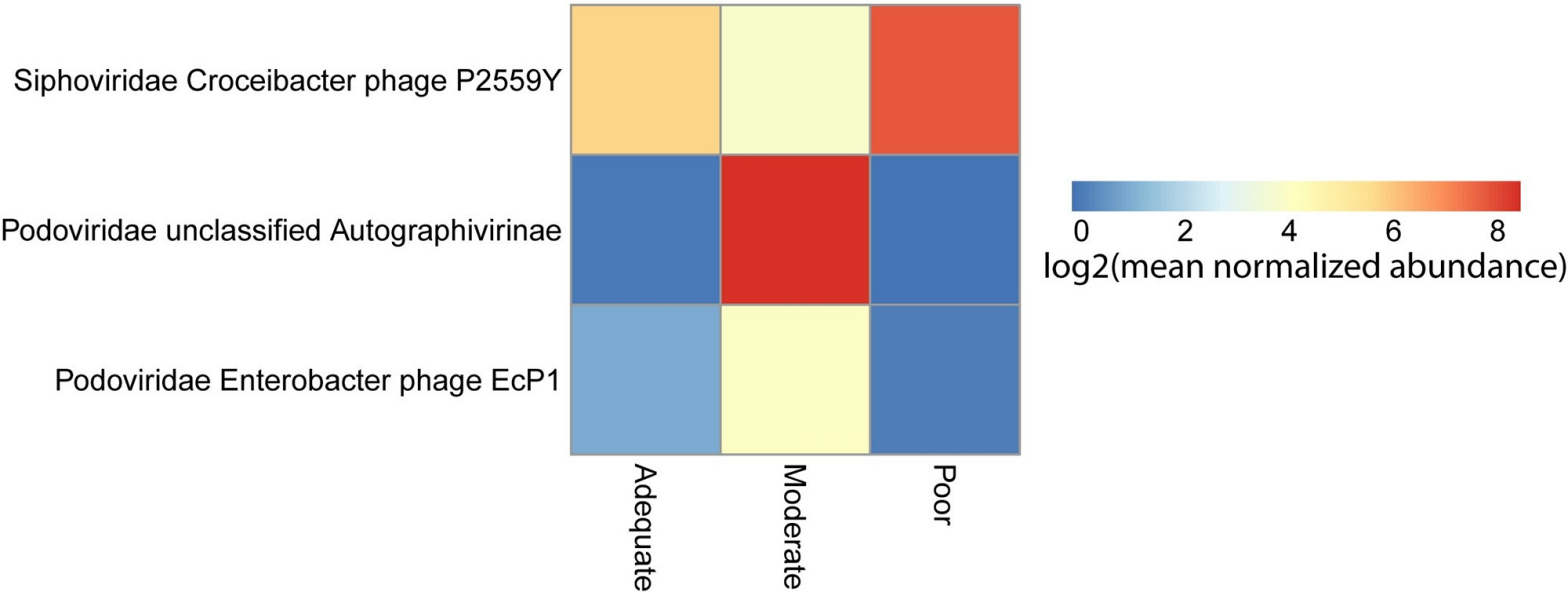
Phages differentially abundant by growth velocity among children with EED. Phage significantly differentially abundant across growth velocity groups colored by log2 mean normalized abundance.

To better understand which bacteriophage might drive the differences in the community structures, we performed a differential abundance analysis. Three phage were significantly differentially abundant between growth velocities (S2 Table and Fig 6). All three differentially abundant phage were *Caudovirales* species.

### Bacteria-bacteriophage interactions differ by growth status

**Fig 7:**
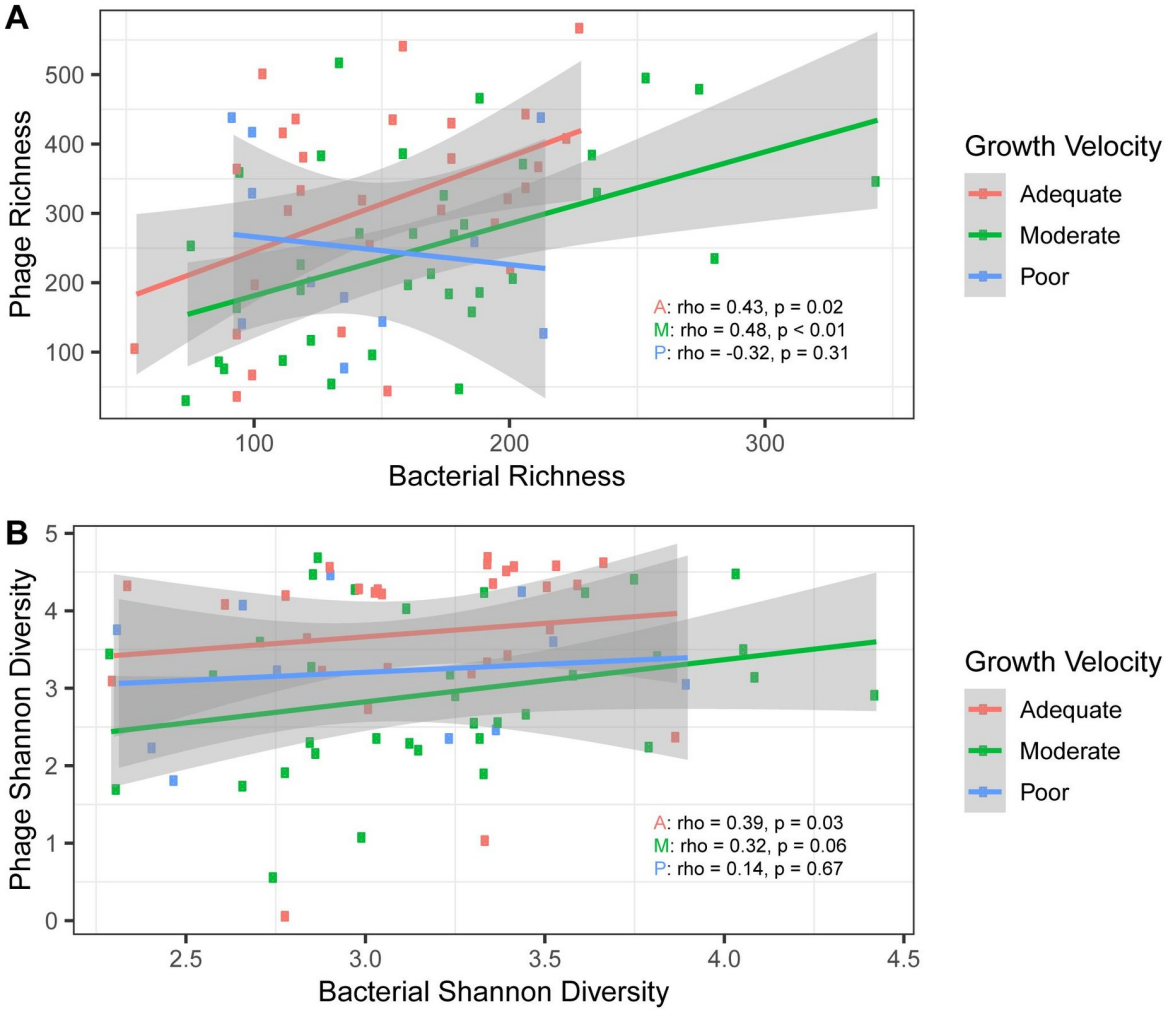
Bacteria-bacteriophage interactions. A) Bacterial richness compared to bacteriophage richness by growth velocity status. B) Bacterial Shannon diversity compared to bacteriophage Shannon diversity. 95% confidence intervals are shown.

To determine the relationship between bacteriophage and bacterial populations in the cohort, we examined the correlation of phage and bacterial richness and phage and bacterial Shannon diversity. Interestingly, we observed that in samples from children with adequate and moderate growth, the bacteriophage richness increased in tandem with bacterial richness (rho = 0.43, p = 0.02 and rho = 0.48, p < 0.01). In contrast, in samples from children with subsequent poor growth, as bacterial richness increased bacteriophage richness did not increase. Furthermore, in samples from children with subsequent poor growth there appears to be a negative correlation between bacterial richness and bacteriophage richness, although this did not reach statistical significance (rho = -0.32, p = 0.31) (Fig 7A). ANCOVA was used to determine if differences in the relationship between bacterial and bacteriophage richness could be explained by growth velocity group while accounting for repeated sampling. While bacterial richness and phage richness had a significant relationship (p = <0.001), as well as growth velocity group and phage richness (p = 0.041), the interaction of bacterial richness and growth velocity group did not have a significant relationship with phage richness (p = 0.14). This suggests that factors other than growth velocity may be important to the differences seen in the correlation of bacteria and phage.

While the bacteriophage richness decreases as bacterial richness increases in samples from children with subsequent poor growth, the Shannon diversity of bacteriophage is stable as bacterial diversity increases (Fig 7B). We next examined pairwise correlations of the differentially abundant bacteriophage and bacterial ASVs (S1 Fig). Notably, there are some positively correlated relationships between differentially abundant bacteriophage and bacterial taxa in samples from children with subsequent poor growth which are negatively correlated in samples from children with subsequent adequate growth (i.e. Podoviridae Enterobacter phage and *Treponema*).

## Discussion

Despite our emerging appreciation of the importance of EED and stunting in global child health, we have a very poor understanding of the mechanisms underlying these inter-related processes. While it is plausible that EED and subsequent stunting are driven (or attenuated) by gut microbes, studies have failed to identify specific responsible taxa, and lack comprehensive assessments of the viral component of the gut community. Combining longitudinal assessment of the gut bacterial and viral communities, percent excretion of an orally administered sugar (lactulose) as a surrogate for enteropathy, and serial anthropometric measurements, we found no difference in the bacterial community structure across the three different growth velocity groups. While we found no difference in the bacterial community structure across the three different growth velocity groups, we did find thirty ASVs differentially associated with growth. Notably, 10 taxa were present only in samples from children with adequate and moderate growth and were completely absent in samples from children with poor growth. Our inability to link poor growth and enteropathy with bacterial community structure is consistent with a cross sectional study of African children [38] and a longitudinal study of children in South India [14] in which bacterial richness and diversity were not affected by stunting.

The presence of several bacterial species only in children with desirable growth warrants comment. Mucosal-associated Helicobacter spp. can elicit the differentiation of CD4^+^ regulatory T cells from peripheral T_reg_ cells in a murine system [39]. Similarly, cluster XIVa *Clostridia* induce T_reg_ function and density in a murine model of colitis [40], and a cocktail of Clostridia from humans, which included *Clostridium* cluster XIVa, attenuated murine colitis and allergic diarrhea, also via T_reg_ induction [41]. *Prevotella histicola* increases tolerogenic responses (including expansion of CD4^+^FoxP3^+^Tregs in the spleens and mesenteric lymph nodes of mice in which experimental autoimmune encephalitis (a model of human multiple sclerosis) is attenuated [42]. *B*. *ruminantium* is one of seven *Bifidobacteria* from infants that express activity against bacterial enteric pathogens in vitro [43]. These experimental data lend credence to our association of these organisms with healthy human growth, and point toward a possible immune suppression effect of these pathobionts.

Our virome data are novel, as we are unaware of any prior study examining the role of viral communities on linear growth in the setting of increased intestinal permeability. In contrast to our prior study of North American birth cohort of younger children [30], viral richness was stable with age in this Malawian cohort. This finding recapitulates findings of a study of the DNA virome of Malawian twins with severe acute malnutrition [36]. While we detected many eukaryotic viruses in these samples, neither the total number of eukaryotic families or a specific viral family were statistically associated with improved linear growth. These finding are similar to a PCR based study examining targeted eukaryotic viruses in children <2 years old with and without diarrhea obtained during the Etiology, Risk Factors, and Interactions of Enteric Infections and Malnutrition and the Consequences for Child Health and Development (MAL-ED) multisite cohort study [44], which found no association with linear growth.

By extending our analysis to the phageome, however, we were able to identify a viral signature related to growth in children with increased intestinal permeability. We identified 3 differentially abundant phage between adequate and poor growth velocities. Due to the more limited understanding of bacteriophage biology, currently not all of the bacterial hosts or range of bacterial hosts for these bacteriophages are known. Stools from children with subsequent adequate growth had a more diverse phageome than children with moderate growth velocity. Bacteriophage richness, however, did not significantly differ between these groups, indicating that this increased diversity is driven by a community with more even representation of its members. However, this increased diversity was only seen in samples prior to the first growth interval, so the biological significance of this finding is unclear.

Because the bacterial and bacteriophage communities are interdependent, we sought to better understand the bacteria-bacteriophage interactions that might be occurring. In children with adequate and moderate growth velocities, i.e., the more favorable patterns, bacterial richness and bacteriophage richness are positively correlated. However, in children with poor growth, as bacterial richness increased, bacteriophage richness did not increase. This suggests that a disruption in the equilibrium of these communities may be associated with subsequent poor growth.

Additionally, our data bring a more comprehensive understanding of gut microbial ecology in children with EED. As we were limited by a small number of samples from children without increased intestinal permeability, we limited our analysis to samples from children with increased permeability. It would be ideal to link attributable risks for stunting to identifiable indices of host factors (e.g., inflammation, gut permeability, specific transported nutrients). We used %L excretion based on earlier work in Weisz et al [25], but recognize that this metric might speak for only a subset of the risk of stunting. However, it is a test that is specific to gut pathobiology. We look forward to future research that might more comprehensively capture the spectrum of physiology that underlies EED-associated stunting. We recognize that EED is a condition of the small intestine, as are alterations in %L, but the specimens sequenced were fecal, and thus an indirect assessment of the microbiota of small intestine. It is well known that individuals from different geographies harbor different communities of gut bacteria and viruses, thus our findings should be considered characteristic only for rural African children with increased intestinal permeability. We additionally note that our study is limited by a relatively small sample size especially with the repeated sampling design of the cohort and the dynamic nature of EED, the bacterial microbiome and virome. Nonetheless, our data do demonstrate associations between specific bacterial and viral taxa and growth velocity, though we cannot yet prove a causal relationship exists between these measures. Our findings demonstrate that including viral communities in future studies of EED and stunting is critical. Otherwise, the microbial ecology of these poorly understood entities is woefully incomplete.

## Supporting information

S1 Fig Heatmap of Spearman correlation between differentially abundant bacteriophage and bacterial ASVs. (TIF)

S1 Checklist STROBE checklist. (DOC)

S1 Table Differentially abundant bacterial taxa identified by DESeq2. (XLSX)

S2 Table Differentially abundant phage identified by DESeq2. (XLSX)
