# Supplementary figures and images for: Growth velocity in children with Environmental Enteric Dysfunction is associated with specific bacterial and viral taxa of the gastrointestinal tract in Malawian children
Source: PLoS Negl Trop Dis. 2020 Jun 23;14(6):e0008387. doi: 10.1371/journal.pntd.0008387 (PMC7310680; doi:10.1371/journal.pntd.0008387)

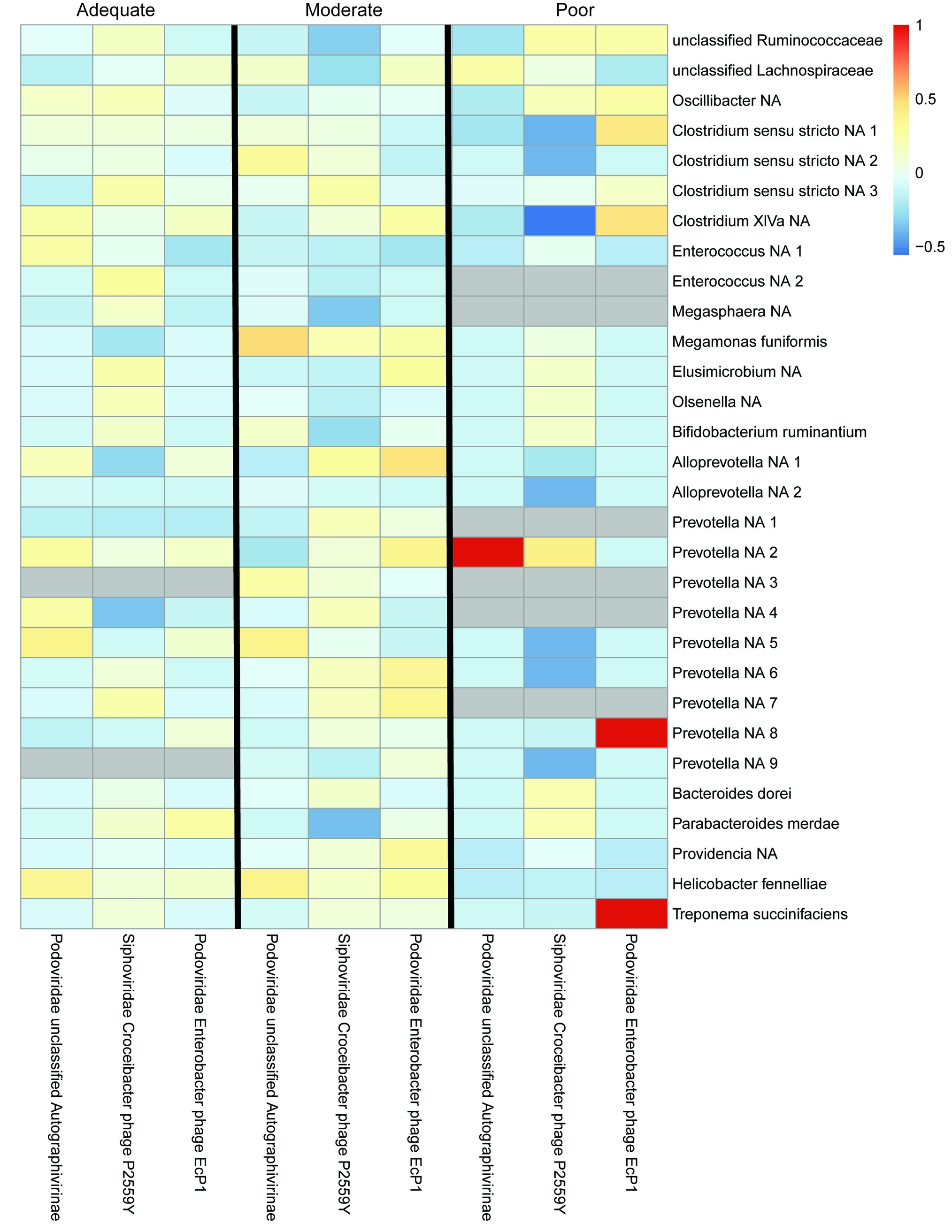

Supplement: S1 Fig — (TIF) [file pntd.0008387.s001.tif]
