# Supplementary material for: Growth velocity in children with Environmental Enteric Dysfunction is associated with specific bacterial and viral taxa of the gastrointestinal tract in Malawian children
Source: PLoS Negl Trop Dis. 2020 Jun 23;14(6):e0008387. doi: 10.1371/journal.pntd.0008387 (PMC7310680; doi:10.1371/journal.pntd.0008387)
Supplement: S1 Checklist — (DOC) [file pntd.0008387.s002.doc]

STROBE Statement—Checklist of items that should be included in reports of ***cohort studies***

|  | Item No | Recommendation |
| --- | --- | --- |
| **Title and abstract** | 1 | (*a*) Indicate the study’s design with a commonly used term in the title or the abstract  Abstract line 25-28 |
|  |  | (*b*) Provide in the abstract an informative and balanced summary of what was done and what was found  Abstract line 25-37 |
| Introduction | | |
| Background/rationale | 2 | Explain the scientific background and rationale for the investigation being reported  Introduction line 64-98 |
| Objectives | 3 | State specific objectives, including any prespecified hypotheses  Introduction line 100-106 |
| Methods | | |
| Study design | 4 | Present key elements of study design early in the paper  Methods line 110-133 |
| Setting | 5 | Describe the setting, locations, and relevant dates, including periods of recruitment, exposure, follow-up, and data collection  Methods line 110-133 |
| Participants | 6 | (*a*) Give the eligibility criteria, and the sources and methods of selection of participants. Describe methods of follow-up  Methods line 112-124 |
|  |  |  |
| Variables | 7 | Clearly define all outcomes, exposures, predictors, potential confounders, and effect modifiers. Give diagnostic criteria, if applicable  Methods line 126-133 |
| Data sources/ measurement | 8* | For each variable of interest, give sources of data and details of methods of assessment (measurement). Describe comparability of assessment methods if there is more than one group  Methods line 119-205 |
| Bias | 9 | Describe any efforts to address potential sources of bias  Methods line 112-117 |
| Study size | 10 | Explain how the study size was arrived at  Methods line 112-113 |
| Quantitative variables | 11 | Explain how quantitative variables were handled in the analyses. If applicable, describe which groupings were chosen and why  Methods lines 126-205 |
| Statistical methods | 12 | (*a*) Describe all statistical methods, including those used to control for confounding  Methods lines 159-163; 195-198; 208-214 |
|  |  | (*b*) Describe any methods used to examine subgroups and interactions  Methods lines 159-163; 195-198; |
|  |  | (*c*) Explain how missing data were addressed. N/A |
|  |  | (*d*) If applicable, explain how loss to follow-up was addressed N/A |
|  |  | (*e*) Describe any sensitivity analyses N/A |
| Results | | |
| Participants | 13* | (a) Report numbers of individuals at each stage of study—eg numbers potentially eligible, examined for eligibility, confirmed eligible, included in the study, completing follow-up, and analysed |
|  |  | (b) Give reasons for non-participation at each stage |
|  |  | (c) Consider use of a flow diagram |
| Descriptive data | 14* | (a) Give characteristics of study participants (eg demographic, clinical, social) and information on exposures and potential confounders  Results line 218-234 and Table 1 |
|  |  | (b) Indicate number of participants with missing data for each variable of interest  NA |
|  |  | (c) Summarise follow-up time (eg, average and total amount)  Results line 218-234 and Table 1 |
| Outcome data | 15* | Report numbers of outcome events or summary measures over time  Results 218-351 |
| Main results | 16 | (*a*) Give unadjusted estimates and, if applicable, confounder-adjusted estimates and their precision (eg, 95% confidence interval). Make clear which confounders were adjusted for and why they were included  NA |
|  |  | (*b*) Report category boundaries when continuous variables were categorized  Methods line 126-133 |
|  |  | (*c*) If relevant, consider translating estimates of relative risk into absolute risk for a meaningful time period  NA |
| Other analyses | 17 | Report other analyses done—eg analyses of subgroups and interactions, and sensitivity analyses  NA |
| Discussion | | |
| Key results | 18 | Summarise key results with reference to study objectives  Discussion lines 363-418 |
| Limitations | 19 | Discuss limitations of the study, taking into account sources of potential bias or imprecision. Discuss both direction and magnitude of any potential bias  Discussion lines 422-429 |
| Interpretation | 20 | Give a cautious overall interpretation of results considering objectives, limitations, multiplicity of analyses, results from similar studies, and other relevant evidence  Discussion lines 359-431 |
| Generalisability | 21 | Discuss the generalisability (external validity) of the study results  Discussion lines 425-431 |
| Other information | | |
| Funding | 22 | Give the source of funding and the role of the funders for the present study and, if applicable, for the original study on which the present article is based  Lines 434-437 |

*Give information separately for exposed and unexposed groups.

**Note:** An Explanation and Elaboration article discusses each checklist item and gives methodological background and published examples of transparent reporting. The STROBE checklist is best used in conjunction with this article (freely available on the Web sites of PLoS Medicine at http://www.plosmedicine.org/, Annals of Internal Medicine at http://www.annals.org/, and Epidemiology at http://www.epidem.com/). Information on the STROBE Initiative is available at http://www.strobe-statement.org.
